# ncRNADrug: a database for validated and predicted ncRNAs associated with drug resistance and targeted by drugs

**DOI:** 10.1093/nar/gkad1042

**Published:** 2023-11-11

**Authors:** Xinyu Cao, Xu Zhou, Fei Hou, Yu-e Huang, Mengqin Yuan, Min Long, Sina Chen, Wanyue Lei, Jicun Zhu, Jiahao Chen, Tao Zhang, An-Yuan Guo, Wei Jiang

**Affiliations:** Fujian Key Laboratory of Precision Medicine for Cancer, the First Affiliated Hospital, Fujian Medical University, Fuzhou 350005, China; Department of Biomedical Engineering, Nanjing University of Aeronautics and Astronautics, Nanjing 211106, China; Department of Biomedical Engineering, Nanjing University of Aeronautics and Astronautics, Nanjing 211106, China; Department of Biomedical Engineering, Nanjing University of Aeronautics and Astronautics, Nanjing 211106, China; Department of Biomedical Engineering, Nanjing University of Aeronautics and Astronautics, Nanjing 211106, China; Department of Biomedical Engineering, Nanjing University of Aeronautics and Astronautics, Nanjing 211106, China; Department of Biomedical Engineering, Nanjing University of Aeronautics and Astronautics, Nanjing 211106, China; Department of Biomedical Engineering, Nanjing University of Aeronautics and Astronautics, Nanjing 211106, China; Department of Biomedical Engineering, Nanjing University of Aeronautics and Astronautics, Nanjing 211106, China; Department of Biomedical Engineering, Nanjing University of Aeronautics and Astronautics, Nanjing 211106, China; Department of Biomedical Engineering, Nanjing University of Aeronautics and Astronautics, Nanjing 211106, China; Department of Biomedical Engineering, Nanjing University of Aeronautics and Astronautics, Nanjing 211106, China; Department of Thoracic Surgery and West China Biomedical Big Data Center, West China Hospital, Sichuan University, Chengdu 610041, China; Fujian Key Laboratory of Precision Medicine for Cancer, the First Affiliated Hospital, Fujian Medical University, Fuzhou 350005, China

## Abstract

Drug resistance is a major barrier in cancer treatment and anticancer drug development. Growing evidence indicates that non-coding RNAs (ncRNAs), especially microRNAs (miRNAs), long non-coding RNAs (lncRNAs) and circular RNAs (circRNAs), play pivotal roles in cancer progression, therapy, and drug resistance. Furthermore, ncRNAs have been proven to be promising novel therapeutic targets for cancer treatment. Reversing dysregulated ncRNAs by drugs holds significant potential as an effective therapeutic strategy for overcoming drug resistance. Therefore, we developed ncRNADrug, an integrated and comprehensive resource that records manually curated and computationally predicted ncRNAs associated with drug resistance, ncRNAs targeted by drugs, as well as potential drug combinations for the treatment of resistant cancer. Currently, ncRNADrug collects 29 551 experimentally validated entries involving 9195 ncRNAs (2248 miRNAs, 4145 lncRNAs and 2802 circRNAs) associated with the drug resistance of 266 drugs, and 32 969 entries involving 10 480 ncRNAs (4338 miRNAs, 6087 lncRNAs and 55 circRNAs) targeted by 965 drugs. In addition, ncRNADrug also contains associations between ncRNAs and drugs predicted from ncRNA expression profiles by differential expression analysis. Altogether, ncRNADrug surpasses the existing related databases in both data volume and functionality. It will be a useful resource for drug development and cancer treatment. ncRNADrug is available at http://www.jianglab.cn/ncRNADrug.

## Graphical Abstract

**Graphical Abstract:**
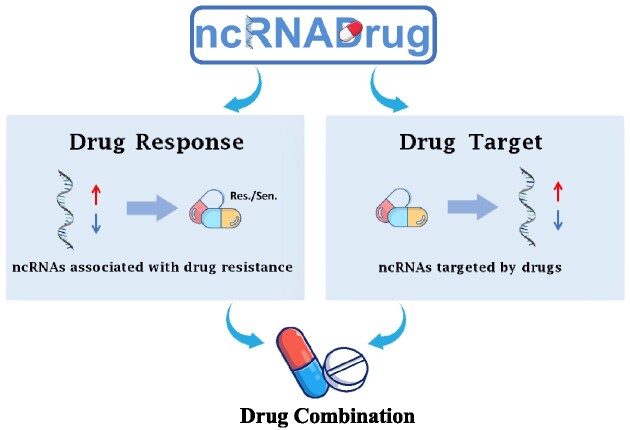

## Introduction

Cancer is a leading cause of death worldwide, posing a serious and long-term threat to human health (1). Chemotherapy continues to serve as the first-line therapeutic approach for cancer patients. However, the emergence of drug resistance significantly impacts the efficacy of chemotherapy, and ultimately leads to treatment failure and recurrence. Cancer cells develop resistance to drugs through various mechanisms, such as drugs efflux, DNA repair and so on (2).

More and more evidence has been proved that non-coding RNAs (ncRNAs), especially microRNAs (miRNAs), long non-coding RNAs (lncRNAs) and circular RNAs (circRNAs), play crucial roles in regulating the occurrence and progression of various cancers, and are closely associated with drug resistance. For instance, miR-149-3p promotes cisplatin resistance in ovarian cancer by targeting CDKN1A and TIMP2 (3). Also, perturbations of ncRNAs can directly or indirectly influence downstream targets and signaling pathways, making ncRNAs as a new class of drug targets. For instance, Sorafenib mediates the dysregulation of the lncRNAs GAS5, HOTTIP and HOXA-AS2 in a panel of human cancer cell lines (4).

Due to the importance of ncRNAs in regulating drug resistance, multiple databases collecting associations between ncRNAs and drugs have been developed, including our previously developed SM2miR (5), D-lnc (6) and ncDR (7), and others, such as miREnvironment (8) and NoncoRNA (9). However, these databases tend to focus primarily on one type of association, either drug response or drug target. There is no database that systematically integrates these two types of associations with up-to-date data. Therefore, it is necessary and highly desirable to construct a centralized resource of ncRNAs associated with drug resistance, ncRNAs targeted by drugs, and potential drug combinations for the treatment of resistant cancer. To fill this gap, we developed ncRNADrug, which collected curated and predicted associations of ncRNAs and drugs.

## Materials and methods

### Data collection

First, we conducted a comprehensive search in the PubMed database using a list of keywords, such as ‘drug and lncRNA’, ‘drug response and circRNA’ and others (Supplementary Table S1, Supplementary Table S2). Subsequently, we screened the literature by reading abstracts, retaining only those related publications with clinical or experimental evidence. Second, we manually extracted the detailed information of experimentally validated ncRNA and drug associations from more than 9000 published papers. The detailed information including ncRNA information (name, ID, type), drug information (name, DrugBank ID, PubChem CID, FDA approved or not), pattern (up-/down-regulated or resistant/sensitive), ncRNA target and pathway, experimental technique (e.g. qRT-PCR, microarray, and RNA-seq), confidence of experiment (low- or high-throughput), species, experimental sample (cell line and/or tissue), phenotype, evidence and reference (PubMed ID, title, published year). In addition, the information in our SM2miR, D-lnc and ncDR were also integrated into the ncRNADrug database. Third, the information about ncRNAs, drugs and phenotypes was further standardized. miRNAs were mapped to miRBase (miRBase Accession). lncRNAs were mapped to Ensembl (Ensembl Gene ID) and NONCODE (NONCODE GENE ID). circRNAs were mapped to circBase (circRNA ID). Drugs were mapped to DrugBank (DrugBank Accession Number), PubChem (PubChem CID) and DTP/NCI (NSC Number). Cancer names were unified as the definition in TCGA.

To predict ncRNAs associated with drug resistance, we firstly obtained ncRNA expression profiles in drug resistant/sensitive tissues and cell lines from GEO database. Also, miRNA and lncRNA expression profiles, as well as drug response data were extracted from NCI60 (10), TANRIC (11), GDSC (12) and CCLE projects (13). Data statistics are shown in Supplementary Table S3. Next, we performed differential expression analysis of ncRNAs between drug resistant and sensitive samples. The differentially expressed ncRNAs were considered as the potential ncRNAs associated with drug resistance.

To predict ncRNAs targeted by drugs, we firstly obtained drug-perturbed ncRNA expression profiles from GEO and Connectivity Map (CMap) databases. Data statistics are shown in Supplementary Table S3. Next, differentially expressed ncRNAs under drug perturbation were identified as the potential drug target ncRNAs.

### Data processing for prediction of ncRNAs associated with drug response

*GEO*. (i) Obtain dataset. We searched all series in the GEO database using the following combination of keywords: (‘drug resistance’ OR ‘drug sensitive’ OR ‘drug response’) AND (‘miRNA’ OR ‘lncRNA’ OR ‘circRNA’). Filter criteria as follows: Study type: ‘Non-coding RNA profiling by array’, ‘Non-coding RNA profiling by genome tiling array’ and ‘Non-coding RNA profiling by high throughput sequencing’. Species: ‘Homo sapiens’, ‘Rattus norvegicus’ and ‘Mus musculus’. (ii) Data Preprocess. Remove probes without ncRNA names. For lncRNA series that do not have GPL annotation, we mapped the probes to the human genome (GRCh38.p13) by the SeqMap tool (1.0.13), and used GENCODE (Release 43) to determine lncRNA genes. If one probe corresponds to multiple ncRNAs, it will be directly abandoned. If an ncRNA has multiple probes, take the average of the expression values of all probes. (iii) Differential expression analysis. For series without biological repeats, calculate the fold change directly by resistant/sensitive; For series with biological repeats: (a) Expression profiling by RT-PCR and microarray data are analyzed by Limma; (b) RNA-seq data with raw count are analyzed by DESeq2; (c) RNA-seq data with normalized data (like TPM, FPKM) are analyzed by Limma. The threshold of significantly differentially expressed ncRNAs: *P*-value < 0.05 and |log_2_(fold change)| > 1.

*NCI60*. (i) Cancer cell line data. The normalized IC50 values (defined as compound concentrations that were required for 50% growth inhibition) across 60 cancer cell lines for 20 218 compounds were obtained from the CellMiner database. Meanwhile, the expression levels of 260 miRNAs of 60 cancer cell lines were acquired and analyzed. (ii) Resistant and sensitive cell lines. For each compound, cell lines with at least 0.8*standard deviation (SD) above the mean normalized IC50 value were defined as resistant to the compound, whereas those with at least 0.8*SD below the mean normalized IC50 value were regarded as sensitive. (iii) Predicted drug resistance-associated miRNAs. For each compound, the significantly differentially expressed miRNAs between the resistant and sensitive cell lines were filtered as drug resistance-related miRNAs, which were computed with the *t*-test (*P*-value < 0.05 and |log_2_(fold change)| > 1).

*GDSC and TANRIC*. (i) Cancer cell line data. The drug response (totally including 135 compounds across 707 cell lines) and lncRNA expression profiles (measuring the RPKM values of 12 727 lncRNAs in 739 cell lines across 20 tumor types) were gained from GDSC and TANRIC (RNA-seq data from CCLE) project, respectively. (ii) Resistant and sensitive cell lines. For each compound, cell lines with at least 0.8*standard deviation above the mean normalized IC50 value were defined as resistant to the compound, whereas those with at least 0.8*SD below the mean normalized IC50 value were regarded as sensitive. (iii) Predicted drug resistance-associated lncRNAs. For one drug in a specific cancer type, if it comprises only one cell in sensitive or resistant class, we applied ‘fold change’ method to measure the extent of association between drug resistance and lncRNAs (|log_2_(fold change)| > 1). Apart from this condition, T-test was used to screen the differentially expressed lncRNAs based on RPKM values between the resistant and sensitive cell lines (*P*-value < 0.05 and |log_2_(fold change)| > 1).

### Data processing for prediction of ncRNAs targeted by drug

*GEO*. (i) Obtain dataset. We searched all series in the GEO database using the following combination of keywords: (‘drug’ OR ‘small molecule’ OR ‘compound’) AND (‘miRNA’ OR ‘lncRNA’ OR ‘circRNA’). Filter criteria as follows: Study type: ‘Non-coding RNA profiling by array’, ‘Non-coding RNA profiling by genome tiling array’ and ‘Non-coding RNA profiling by high throughput sequencing’. Species: ‘Homo sapiens’, ‘Rattus norvegicus’ and ‘Mus musculus’. (ii) Data Preprocess. Remove probes without ncRNA names. For lncRNA series that do not have GPL annotation, we mapped the probes to the human genome (GRCh38.p13) by the SeqMap tool (1.0.13), and used GENCODE (Release 43) to determine lncRNA genes. If one probe corresponds to multiple ncRNAs, it will be directly abandoned. If an ncRNA has multiple probes, take the average of the expression values of all probes. (iii) Differential expression analysis. For series without biological repeats, calculate the foldchange directly by treat/control; For series with biological repeats: (a) RT-PCR and microarray data are analyzed by Limma; (b) RNA-seq data with raw count are analyzed by DESeq2; (c) RNA-seq data with normalized data (like TPM, FPKM) are analyzed by Limma. The threshold of significantly differentially expressed ncRNAs: *P*-value < 0.05 and |log_2_(fold change)| > 1.

*CMap*. We retrieved 6100 drug-perturbed gene expression datasets from CMap. Then we re-annotated the probes to lncRNAs. The differentially expressed lncRNAs between drug-treated samples and control samples were considered as drug-affected lncRNAs. Here, two-fold change was used for the identification of differentially expressed lncRNAs.

### Database design and implementation

ncRNADrug was constructed based on Apache Tomcat (https://tomcat.apache.org/) and MySQL (https://www.mysql.com/). Web interfaces were developed by PHP (https://www.php.net/), HTML5, CSS2, JQuery and BootStrap. Highcharts (https://www.highcharts.com/) and cytoscape.js (https://js.cytoscape.org/) were adopted to generate interactive graphs. DataTables (https://datatables.net/) was used to build interactive data tables.

## Results

### Data statistics in ncRNADrug

**Figure 1.**
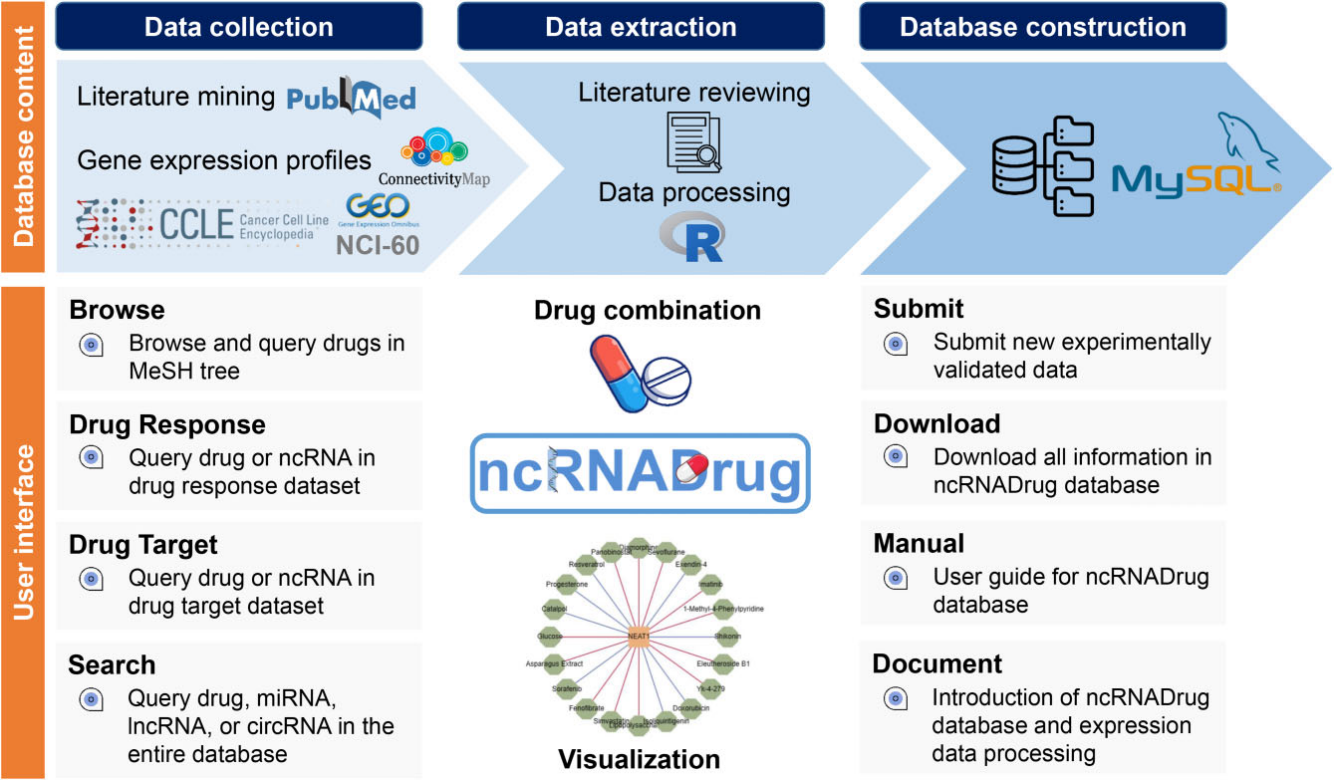
Overview of ncRNADrug database.

An overview of ncRNADrug database is shown in Figure 1. ncRNADrug provides a user-friendly, open access web interface for searching, browsing and downloading data. So far, in terms of experimentally validated entries, ncRNADrug contains 29 551 entries involving 9195 ncRNAs (2248 miRNAs, 4145 lncRNAs and 2802 circRNAs) associated with the drug resistance of 266 drugs, and 32 969 entries involving 10 480 ncRNAs (4338 miRNAs, 6087 lncRNAs and 55 circRNAs) targeted by 965 drugs. Further analysis revealed that the associations between ncRNAs and drugs could vary based on specific conditions, such as different diseases, cell lines, and species. For example, lncRNA CRNDE promotes colorectal cancer cell resistance to oxaliplatin, whereas it attenuates resistance to oxaliplatin in gastric cancer. Dexamethasone can up-regulate lncRNA GAS5 expression in prostate cancer, whereas it down-regulated GAS5 expression in diabetes. In terms of predicted entries, ncRNADrug contains 624 246 entries involving 134 201 ncRNAs (3601 miRNAs, 32 892 lncRNAs and 97 708 circRNAs) associated with the drug resistance of 5588 drugs, and 285 100 predicted entries involving 61 602 ncRNAs (5423 miRNAs, 36 814 lncRNAs and 19 365 circRNAs) targeted by 1303 drugs. Table 1 and Figures S1, S2 summarized the results deposited in ncRNADrug.

**Table 1.** Data summary of ncRNAs associated with drug resistance (drug response) and ncRNAs targeted by drugs (drug target) in ncRNADrug

	Drug response				Drug target		
Source	Literature	GEO	NCI60	CCLE	Literature	GEO	CMap
Drug	266	31	5457	135	965	159	1190
miRNA	2248	3601	260	-	4338	5423	-
lncRNA	4145	31 868	-	9000	6087	36 796	129
circRNA	2802	97 708	-	-	55	19 365	-
Association^a^	29 551	284 534	128 965	210 747	32 969	265 154	19 946

^a^The number of drug-ncRNA pairs.

Furthermore, we also compared the amount of experimentally validated datasets and functionality of ncRNADrug with commonly used databases (Table 2 and Figure S3). In terms of drug response, ncDR (7) provides curated and predicted drug resistance-related ncRNAs, but does not include circRNAs. NoncoRNA (9) only provides curated associations, and most of ncRNAs and drugs are covered by ncRNADrug. In terms of drug target, miREnvironment (8) curated and collected experimentally supported miRNA and environmental factor interplays and their associated phenotypes. However, drugs were only a small part of environmental factors included in miREnvironment, and drug information has not been mapped to drugs in standard databases like DrugBank.

**Table 2.** Comparison of curated information about ncRNAs associated with drug resistance (drug response) and ncRNAs targeted by drugs (drug target) in ncRNADrug with other databases

		Drug response			Drug target			
	Database	ncDR	NoncoRNA	**ncRNADrug**	SM2miR	miREnvironment	D-lnc	**ncRNADrug**
Data volume	Drug	145	154	266	255	161	59	965
	miRNA	877	1006	2248	1658	686	−	4338
	lncRNA	162	3599	4145	−	−	4917	6087
	circRNA	−	959	2802	−	−	−	55
Database function	Prediction	√	×	√	×	×	√	√
	Drug combination	×	×	√	×	×	×	√
	ncRNA target and pathway	√	√	√	×	×	×	√

In conclusion, the ncRNADrug database surpasses existing databases of drug and ncRNA associations in both data volume and functionality.

### Web interface and database functions

**Figure 2.**
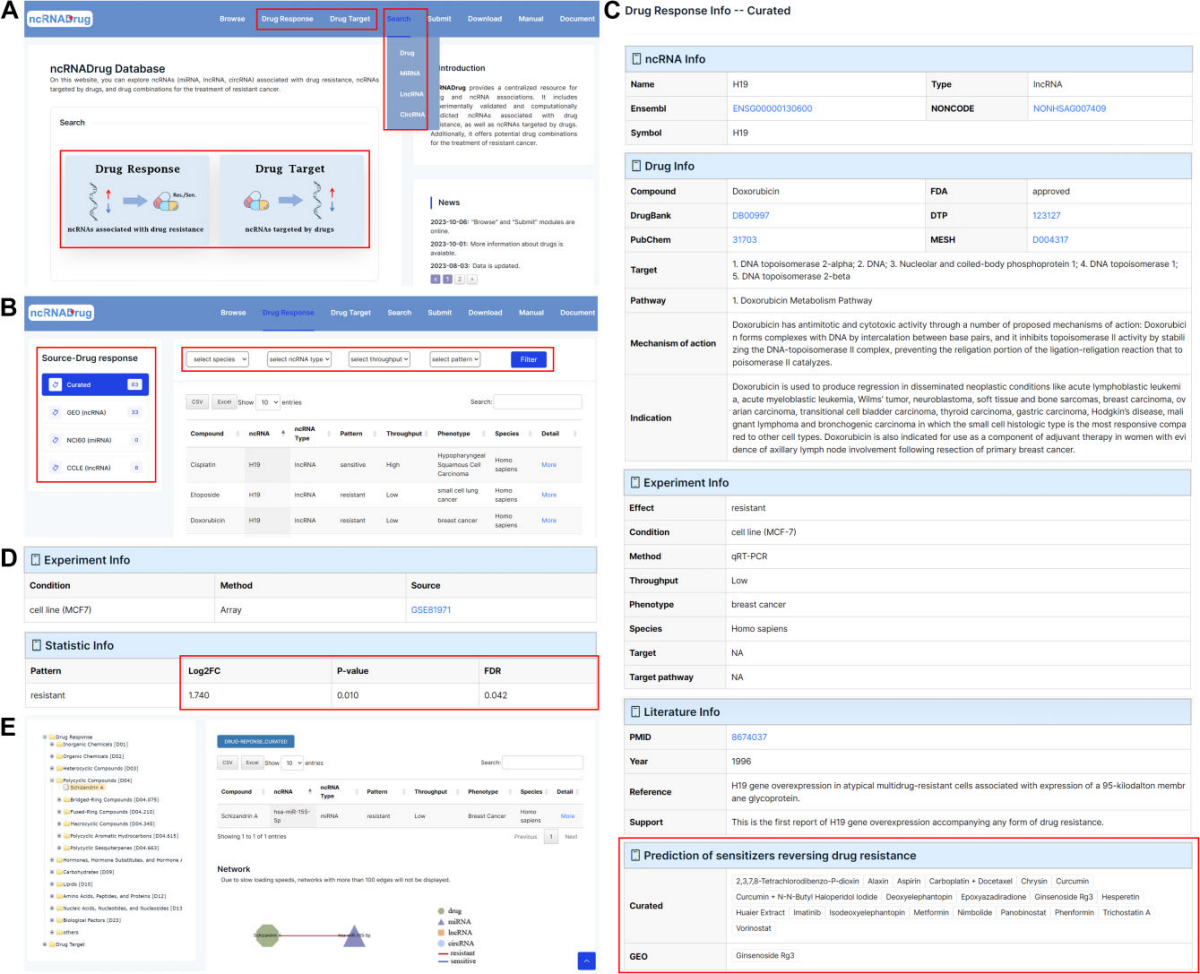
Main functions and usage of ncRNADrug. (**A**) Interface for two query methods: search by ‘Drug Response’ or ‘Drug Target’; search by drug, miRNA, lncRNA, or circRNA. (**B**) The ‘Result’ page. (**C**) The ‘Detail’ page of curated drug response result. (**D**) The ‘Detail’ page of predicted drug response result. (**E**) The ‘Browse’ page.

Users can search for ‘Drug Response’ or ‘Drug Target’ by clicking on the image on the Homepage or the options in the top menu bar. If users pay more attention to specific drug or ncRNA, not just limited to drug response or drug target, it is recommended to search by selecting the ‘Search’ drop-down box in the top menu bar (Figure 2A). On the ‘Search’ page, ncRNADrug allows users to search by drug or ncRNA (miRNA, lncRNA, circRNA). ncRNADrug supports fuzzy search, returning the most relevant results. Users can browse associations from different sources (curated, GEO, CMap, NCI60 and CCLE) in the left sidebar (Figure 2B). Several filters (species, ncRNA type, throughput, expression pattern) are provided at the top, allowing users to further filter based on their specific interests (Figure 2B). For curated associations, ncRNADrug provides four filters: species, ncRNA type, throughput and ncRNA pattern. For predicted associations, users can filter by ncRNA type, ncRNA pattern, foldchange or p-value. A network graph with corresponding ncRNA or drug as central node is provided at the bottom of the ‘Result’ page. Clicking ‘More’ will direct users to a ‘Detail’ page to show detailed information, for curated associations, detailed information consists of the following sections: ncRNA info, Drug info, Experiment info and Literature info (Figure 2C). ncRNADrug also added a widget offering predicted sensitizers that may reverse drug resistance (Figure 2C). Clicking on each drug button will return the corresponding entry. For predicted associations, detailed information comprises the following sections: ncRNA info, Drug info, Experiment info and Statistic info, which includes fold change, p-value and false discovery rate (FDR) (Figure 2D). ‘Browse’ page allows users to browse and query drugs in the curated drug response and drug target datasets in the Medical Subject Headings (MeSH) tree (Figure 2E). ‘Submit’ page allows users to submit new validated data for updating the database. If the records are approved by our review committee, they will be available in ncRNADrug. Users can download all data on the ‘Download’ page, ncRNADrug provides two formats of the downloadable file in TXT and Excel formats, respectively. The detailed data process is available on the ‘Document’ page. A comprehensive manual on how to use ncRNADrug is available on the ‘Manual’ page.

### Application: drug combination

Drug combination is considered as a promising therapy to overcome drug resistance and improve the efficacy of cancer treatment (14). For example, BRCA1 and BRCA2 play key roles in DNA damage repair, PARP inhibitors (PARPi) were considered as possible therapeutic agents for patients with BRCA1/2 germline mutations (15). However, intragenic deletion and genetic reversion of BRCA1/2 has been demonstrated to alter sensitivity of BRCA1/2 mutated cells to PARPis (16). Michal et al showed that micromolar concentrations of vorinostat could lead to a major reduction in the level of BRCA1 and led to increased sensitivity of various cancer cell lines to ABT-888 (a PARPi) (17). In addition, accumulated evidence demonstrates that dysregulated ncRNAs are important regulators in the development of drug resistance (18,19). Thus, we developed a new function to assist researchers in exploring sensitizers that can reverse the dysregulated expression of ncRNAs associated with drug resistance on the ‘Result’ page. Here, we showed how to identify drug-resistant lncRNAs, acquire drugs that influence lncRNA expression, and explore potential drug combinations using the following example, which may aid users in generating new hypotheses for further experimental validation.

If users are interested in lncRNAs related to doxorubicin resistance, they can input ‘doxorubicin’ in the search box on the ‘Drug Response’ page. Then, select ‘Homo sapiens’ in species, ‘lncRNA’ in ncRNA type, and ‘low’ in throughput to filter the results on the ‘Result’ page. We can obtain 145 related associations validated by low-throughput experiments in different conditions. Furthermore, input ‘H19’ in the search box in the upper right corner to focus on a specific lncRNA. We can obtain 5 entries, which show that H19 is up-regulated in doxorubicin resistant cells in several diseases, such as breast cancer (20).

Next, if users want to explore which drugs can reverse the expression of H19 in doxorubicin resistant cells, they can input ‘H19’ in the search box on the ‘Drug Target’ page. Then, select ‘low’ in throughput, and ‘down-regulated’ in pattern to filter the results on the ‘Result’ page. We can obtain 23 validated associations in different species and different conditions, including 20 candidate drugs with potential to down-regulate H19 expression, such as aspirin, curcumin, and metformin.

Finally, sensitizers can be predicted through exploring drugs that can reverse the dysregulated expression of ncRNAs in drug resistance. Thus, the above 20 candidate drugs might be the sensitizers of doxorubicin resistance. Take aspirin as an example, the new hypothesis is that aspirin has the potential to reverse doxorubicin resistant via down-regulating H19 expression. In fact, several studies have reported on the combination of aspirin and doxorubicin. For example, Xie et al found that aspirin enhances the sensitivity of hepatocellular carcinoma (HCC) side population cells to doxorubicin via up-regulating miR-491 and down-regulating target gene ABCG2 (21). miR-491 was demonstrated to be a potential target for the therapy of HCCs, since it can decrease cancer stem cells-like properties of HCC by inhibition of GIT-1/NF-κB-mediated EMT (22). Apoorva et al found that aspirin prevents doxorubicin-induced repression of SMAR1 (scaffold matrix attachment region binding protein 1) and proliferation of breast cancer stem-like cells, consequently enhancing the cytotoxicity of doxorubicin (23). Previous reports have revealed that SMAR1, acting as a potential tumor suppressor, plays a critical role in maintaining genomic stability and cell cycle progression (24).

## Discussion

Studies have increasingly indicated that ncRNAs play crucial roles in biological process and tumorigenesis. High-throughput sequencing has generated extensive data, bringing new insights to researches on ncRNA and opening up new prospects to expand druggable targets from proteins to ncRNAs. In the past few years, several databases have been developed to aid researchers in exploring the associations between ncRNAs and drugs. However, to the best of our knowledge, none of these resources have integrated both experimentally supported drug-resistance related ncRNAs and drug target ncRNAs. Thus, we developed ncRNADrug, a centralized database that encompasses experimentally validated and computationally predicted ncRNAs associated with drug resistance, as well as ncRNAs targeted by drugs. In addition to collecting a great number of experimentally supported associations between ncRNA and drug, the widget within ncRNADrug offers potential drug combinations for the treatment of resistant cancer. These features not only provide mechanistic insights but also supply valuable experimental evidence to inform future drug development endeavors. We believe that ncRNADrug will serve as a valuable resource, contributing to drug discovery and cancer treatment. However, although ncRNADrug surpasses the existing databases in data volume, we must declare that there may be missing some data due to the challenges of capturing all relevant information during manual curation. In the future, we will continuously enhance ncRNADrug by adding more data and functions, ensuring regular updates to improve its efficacy and usefulness.

## Supplementary Material

gkad1042_Supplemental_File

## Data Availability

ncRNADrug is freely available at: http://www.jianglab.cn/ncRNADrug/.

## References

[1] Bray F. , Laversanne M., Weiderpass E., Soerjomataram I. The ever-increasing importance of cancer as a leading cause of premature death worldwide. Cancer. 2021; 127:3029–3030. 34086348 10.1002/cncr.33587

[2] Housman G. , Byler S., Heerboth S., Lapinska K., Longacre M., Snyder N., Sarkar S. Drug resistance in cancer: an overview. Cancers. 2014; 6:1769–1792. 25198391 10.3390/cancers6031769 PMC4190567

[3] Wang J. , Liu L. MiR-149-3p promotes the cisplatin resistance and EMT in ovarian cancer through downregulating TIMP2 and CDKN1A. J. Ovarian Res. 2021; 14:165. 34798882 10.1186/s13048-021-00919-5 PMC8605569

[4] Faranda T. , Grossi I., Manganelli M., Marchina E., Baiocchi G., Portolani N., Crosatti M., De Petro G., Salvi A. Differential expression profiling of long non-coding RNA GAS5 and miR-126-3p in human cancer cells in response to sorafenib. Sci. Rep. 2019; 9:9118. 31235746 10.1038/s41598-019-45604-2 PMC6591391

[5] Liu X. , Wang S., Meng F., Wang J., Zhang Y., Dai E., Yu X., Li X., Jiang W. SM2miR: a database of the experimentally validated small molecules' effects on microRNA expression. Bioinformatics. 2013; 29:409–411. 23220571 10.1093/bioinformatics/bts698

[6] Jiang W. , Qu Y., Yang Q., Ma X., Meng Q., Xu J., Liu X., Wang S. D-lnc: a comprehensive database and analytical platform to dissect the modification of drugs on lncRNA expression. RNA Biol. 2019; 16:1586–1591. 31390943 10.1080/15476286.2019.1649584 PMC6779407

[7] Dai E. , Yang F., Wang J., Zhou X., Song Q., An W., Wang L., Jiang W. ncDR: a comprehensive resource of non-coding RNAs involved in drug resistance. Bioinformatics. 2017; 33:4010–4011. 28961690 10.1093/bioinformatics/btx523

[8] Yang Q. , Qiu C., Yang J., Wu Q., Cui Q. miREnvironment database: providing a bridge for microRNAs, environmental factors and phenotypes. Bioinformatics. 2011; 27:3329–3330. 21984757 10.1093/bioinformatics/btr556

[9] Li L. , Wu P., Wang Z., Meng X., Zha C., Li Z., Qi T., Zhang Y., Han B., Li S. et al. NoncoRNA: a database of experimentally supported non-coding RNAs and drug targets in cancer. J. Hematol. Oncol. 2020; 13:15. 32111231 10.1186/s13045-020-00849-7 PMC7048090

[10] Shoemaker R.H. The NCI60 human tumour cell line anticancer drug screen. Nat. Rev. Cancer. 2006; 6:813–823. 16990858 10.1038/nrc1951

[11] Li J. , Han L., Roebuck P., Diao L., Liu L., Yuan Y., Weinstein J.N., Liang H. TANRIC: an interactive open platform to explore the function of lncRNAs in cancer. Cancer Res. 2015; 75:3728–3737. 26208906 10.1158/0008-5472.CAN-15-0273 PMC4573884

[12] Yang W. , Soares J., Greninger P., Edelman E.J., Lightfoot H., Forbes S., Bindal N., Beare D., Smith J.A., Thompson I.R. et al. Genomics of drug sensitivity in cancer (GDSC): a resource for therapeutic biomarker discovery in cancer cells. Nucleic Acids Res. 2013; 41:D955–D961. 23180760 10.1093/nar/gks1111 PMC3531057

[13] Barretina J. , Caponigro G., Stransky N., Venkatesan K., Margolin A.A., Kim S., Wilson C.J., Lehár J., Kryukov G.V., Sonkin D. et al. The Cancer Cell Line Encyclopedia enables predictive modelling of anticancer drug sensitivity. Nature. 2012; 483:603–607. 22460905 10.1038/nature11003 PMC3320027

[14] Anighoro A. , Bajorath J., Rastelli G. Polypharmacology: challenges and opportunities in drug discovery. J. Med. Chem. 2014; 57:7874–7887. 24946140 10.1021/jm5006463

[15] Farmer H. , McCabe N., Lord C.J., Tutt A.N., Johnson D.A., Richardson T.B., Santarosa M., Dillon K.J., Hickson I., Knights C. et al. Targeting the DNA repair defect in BRCA mutant cells as a therapeutic strategy. Nature. 2005; 434:917–921. 15829967 10.1038/nature03445

[16] Kim Y. , Kim A., Sharip A., Sharip A., Jiang J., Yang Q., Xie Y. Reverse the resistance to PARP inhibitors. Int. J. Biol. Sci. 2017; 13:198–208. 28255272 10.7150/ijbs.17240 PMC5332874

[17] Yalon M. , Tuval-Kochen L., Castel D., Moshe I., Mazal I., Cohen O., Avivi C., Rosenblatt K., Aviel-Ronen S., Schiby G. et al. Overcoming resistance of cancer cells to PARP-1 inhibitors with three different drug combinations. PLoS One. 2016; 11:e0155711. 27196668 10.1371/journal.pone.0155711 PMC4873128

[18] He B. , Zhao Z., Cai Q., Zhang Y., Zhang P., Shi S., Xie H., Peng X., Yin W., Tao Y. et al. miRNA-based biomarkers, therapies, and resistance in Cancer. Int. J. Biol. Sci. 2020; 16:2628–2647. 32792861 10.7150/ijbs.47203 PMC7415433

[19] Chen B. , Dragomir M.P., Yang C., Li Q., Horst D., Calin G.A. Targeting non-coding RNAs to overcome cancer therapy resistance. Signal Transduct. Targeted Ther. 2022; 7:121. 10.1038/s41392-022-00975-3 PMC9008121 35418578

[20] Wang Y. , Zhou P., Li P., Yang F., Gao X.Q. Long non-coding RNA H19 regulates proliferation and doxorubicin resistance in MCF-7 cells by targeting PARP1. Bioengineered. 2020; 11:536–546. 32345117 10.1080/21655979.2020.1761512 PMC8291873

[21] Xie Z.Y. , Liu M.S., Zhang C., Cai P.C., Xiao Z.H., Wang F.F. Aspirin enhances the sensitivity of hepatocellular carcinoma side population cells to doxorubicin via miR-491/ABCG2. Biosci. Rep. 2018; 38:BSR20180854. 30120100 10.1042/BSR20180854 PMC6239265

[22] Yang X. , Ye J., Yan H., Tang Z., Shen J., Zhang J., Yang L. MiR-491 attenuates cancer stem cells-like properties of hepatocellular carcinoma by inhibition of GIT-1/NF-κB-mediated EMT. Tumour Biol. 2016; 37:201–209. 26188902 10.1007/s13277-015-3687-5

[23] Bhattacharya A. , Mukherjee S., Khan P., Banerjee S., Dutta A., Banerjee N., Sengupta D., Basak U., Chakraborty S., Dutta A. et al. SMAR1 repression by pluripotency factors and consequent chemoresistance in breast cancer stem-like cells is reversed by aspirin. Sci. Signal. 2020; 13:eaay6077. 33082288 10.1126/scisignal.aay6077

[24] Paul D. , Ghorai S., Dinesh U.S., Shetty P., Chattopadhyay S., Santra M.K. Cdc20 directs proteasome-mediated degradation of the tumor suppressor SMAR1 in higher grades of cancer through the anaphase promoting complex. Cell Death. Dis. 2017; 8:e2882. 28617439 10.1038/cddis.2017.270 PMC5520925

