# Supplementary material for: ncRNADrug: a database for validated and predicted ncRNAs associated with drug resistance and targeted by drugs
Source: Nucleic Acids Res. 2023 Nov 11;52(D1):D1393–9. doi: 10.1093/nar/gkad1042 (PMC10767907; doi:10.1093/nar/gkad1042)
Supplement: gkad1042_Supplemental_File [file gkad1042_supplemental_file.pdf]

Supplementary Table S1. Keywords for drug response

Drug response	Logical operator	ncRNA	Logical operator	Filter	Logical operator	Filter
drug resistan*		miR-* OR let-7*				
drug sensitiv*	AND	long non-coding RNA<br>lncRNA<br>circular RNA	AND	express*	NOT	Review
drug response		circRNA				

Supplementary Table S2. Keywords for drug target

Drug	Logical operator	ncRNA	Logical operator	Filter	Logical operator	Filter	Logical operator	Filter
drug<br>small molecule<br>compound	AND	miR-* OR let-7*<br>long non-coding RNA<br>lncRNA<br>circular RNA<br>circRNA	AND	regulat*	AND	express*	NOT	Review

Supplementary Table S3. Gene expression datasets for prediction

ncRNA	Drug response			Drug target	
	GEO	NCI60	CCLE	GEO	CMap
miRNA	818	60	-	2366	-
lncRNA	175	-	739	349	6100
circRNA	40	-	-	137	-

Supplementary Figure S1

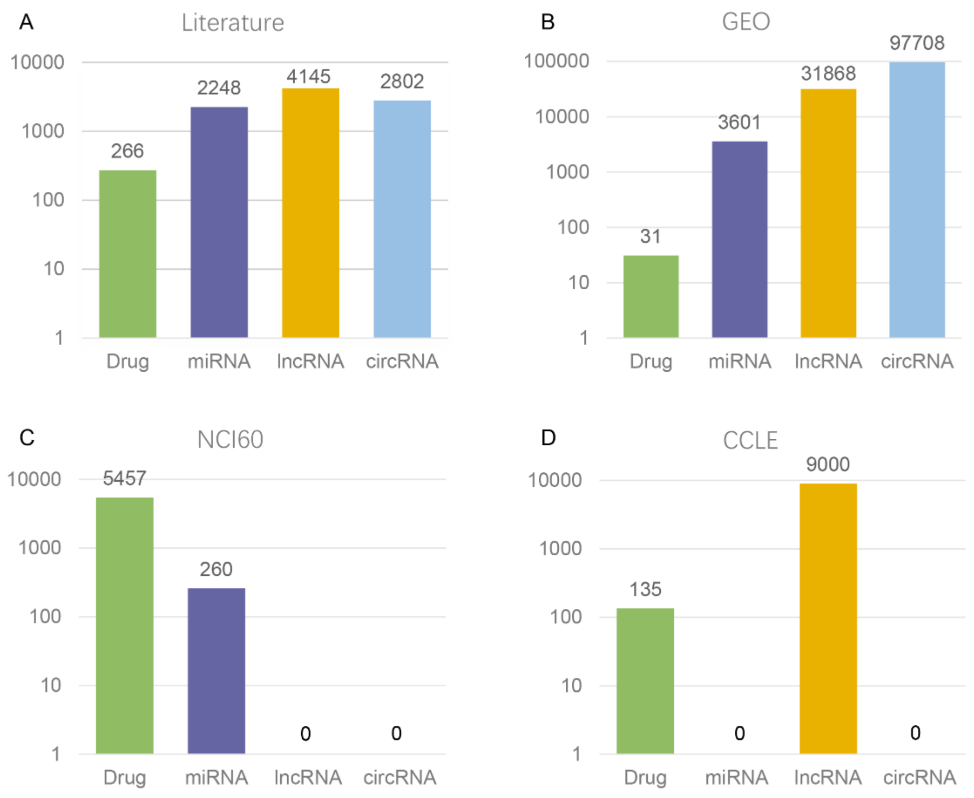

Figure S1. Number of drugs, miRNAs, lncRNAs, and circRNAs related to drug response from different sources, including literature (A), GEO (B), NCI60 (C), and CCLE (D)

Supplementary Figure S2

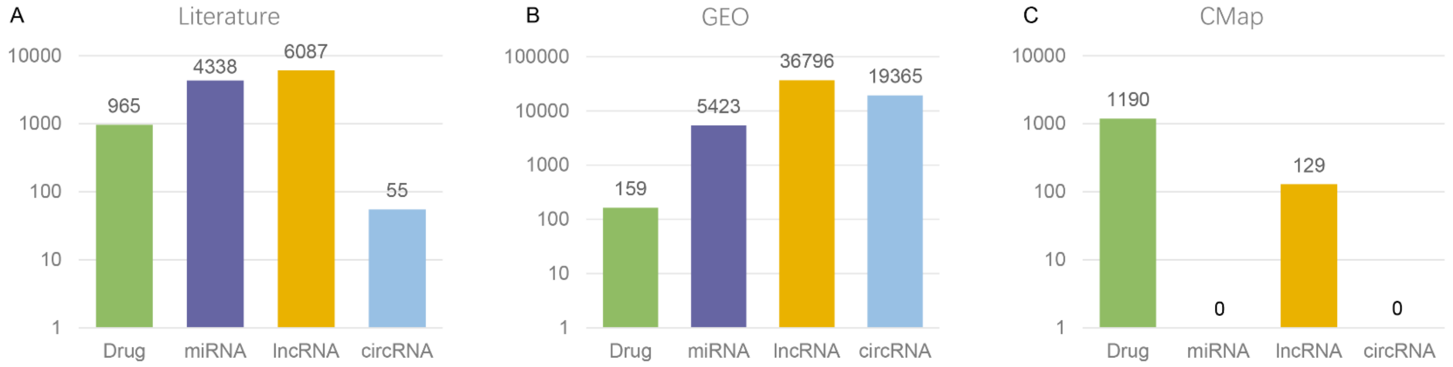

Figure S2. Number of drugs, miRNAs, lncRNAs, and circRNAs related to drug target from different sources, including literature (A), GEO (B), and CMap (C)

Supplementary Figure S3

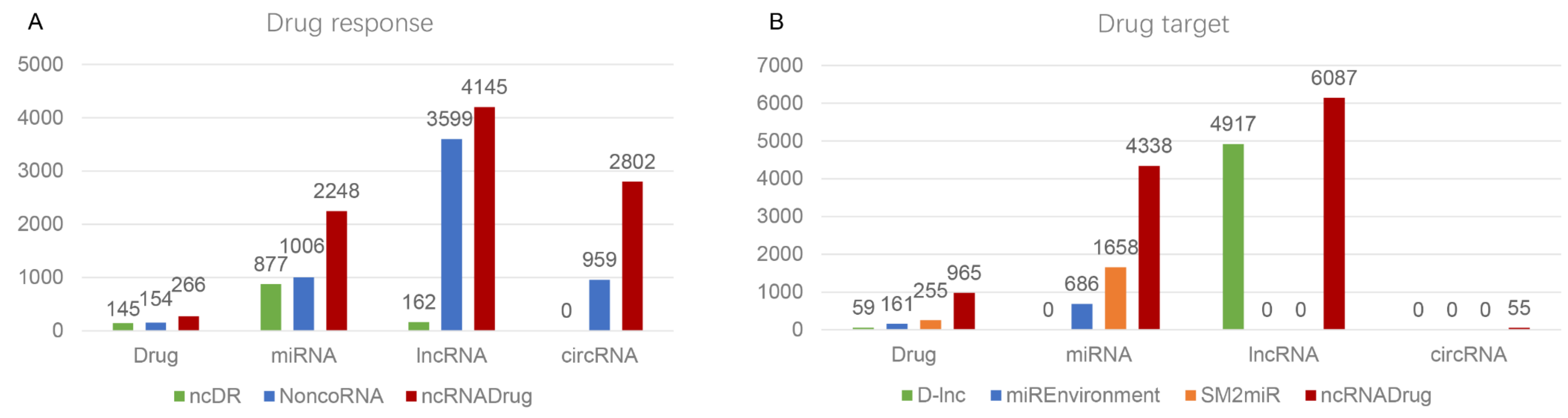

Figure S3. Number of drugs, miRNAs, lncRNAs, and circRNAs related to drug response (A) and drug target (B) from different databases
